# A Machine-Learning Model for the Prognostic Role of C-Reactive Protein in Myocarditis

**DOI:** 10.3390/jcm11237068

**Published:** 2022-11-29

**Authors:** Anna Baritussio, Chun-yan Cheng, Giulia Lorenzoni, Cristina Basso, Stefania Rizzo, Monica De Gaspari, Francesco Fachin, Andrea Silvio Giordani, Honoria Ocagli, Elena Pontara, Maria Grazia Peloso Cattini, Elisa Bison, Nicoletta Gallo, Mario Plebani, Giuseppe Tarantini, Sabino Iliceto, Dario Gregori, Renzo Marcolongo, Alida Linda Patrizia Caforio

**Affiliations:** 1 Cardiology, Department of Cardiac, Thoracic, Vascular Sciences and Public Health, University of Padua, 35128 Padua, Italy; 2 Statistics, Department of Cardiac, Thoracic, Vascular Sciences and Public Health, University of Padua, 35128 Padua, Italy; 3 Cardiac Pathology, Department of Cardiac, Thoracic, Vascular Sciences and Public Health, University of Padua, 35128 Padua, Italy; 4 Department of Laboratory Medicine, University of Padua, 35128 Padua, Italy

**Keywords:** myocarditis, C-reactive protein, endomyocardial biopsy, prognosis

## Abstract

Aims: The role of inflammation markers in myocarditis is unclear. We assessed the diagnostic and prognostic correlates of C-reactive protein (CRP) at diagnosis in patients with myocarditis. Methods and results: We retrospectively enrolled patients with clinically suspected (CS) or biopsy-proven (BP) myocarditis, with available CRP at diagnosis. Clinical, laboratory and imaging data were collected at diagnosis and at follow-up visits. To evaluate predictors of death/heart transplant (Htx), a machine-learning approach based on random forest for survival data was employed. We included 409 patients (74% males, aged 37 ± 15, median follow-up 2.9 years). Abnormal CRP was reported in 288 patients, mainly with CS myocarditis (*p* < 0.001), recent viral infection, shorter symptoms duration (*p* = 0.001), chest pain (*p* < 0.001), better functional class at diagnosis (*p* = 0.018) and higher troponin I values (*p* < 0.001). Death/Htx was reported in 13 patients, of whom 10 had BP myocarditis (overall 10-year survival 94%). Survival rates did not differ according to CRP levels (*p* = 0.23). The strongest survival predictor was LVEF, followed by anti-nuclear auto-antibodies (ANA) and BP status. Conclusions: Raised CRP at diagnosis identifies patients with CS myocarditis and less severe clinical features, but does not contribute to predicting survival. Main death/Htx predictors are reduced LVEF, BP diagnosis and positive ANA.

## 1. Introduction

Myocarditis is an inflammatory disease of the myocardium characterised by the presence of inflammatory infiltrates within the myocardium, myocyte degeneration and necrosis of non-ischaemic origin on endomyocardial biopsy (EMB) [1]. Aetiology of myocardial inflammation in myocarditis is heterogeneous and comprises bacterial, viral, toxic and immune-mediated causes [1,2,3,4]. C-reactive protein (CRP), an acute phase protein and biomarker of systemic inflammation, has been associated with different cardiovascular diseases [5,6,7] and has an established diagnostic and prognostic role in atherosclerosis; less is known about its role in non-ischemic cardiomyopathies. A recent paper suggests that elevated CRP, troponin I and echocardiographic global longitudinal strain in combination with troponin I predict endomyocardial biopsy-proven inflammatory cardiomyopathy [8]. We aimed to assess CRP in patients with clinically suspected and biopsy-proven myocarditis and its clinical, laboratory and imaging correlates, and to explore its potential role as a prognostic biomarker. In the last ten years, the use of machine learning (ML) approaches for developing predictive models within clinical studies has been growing, especially in the field of cardiology [9,10,11,12,13]. In our study we used a random forest (RF) algorithm. As well as the improvement in predictive capability of the model, an important reason for having chosen the RF algorithm is to identify survival predictors even in the presence of low number of events at follow-up. In such situations, the estimation of more traditional multivariable Cox proportional hazard models is not recommended, in particular when the goal of the study is to explore potential prognostic features.

## 2. Materials and Methods

We retrospectively analysed our registry of 843 patients with clinically suspected or biopsy proven myocarditis, admitted to our institution, a tertiary referral centre, from January 1992 to July 2020, and regularly followed-up at our cardio-immunology outpatient clinic. Myocarditis was defined according to the 2013 European Society of Cardiology (ESC) consensus [1]: in the absence of histological evidence, a clinically suspected myocarditis should be diagnosed in the presence of one or more of the clinical presentations (acute coronary syndrome-like; new, worsening or chronic heart failure; life-threatening arrhythmias and/or cardiogenic shock) and one or more of the diagnostic criteria from different categories (ECG/Holter monitoring; elevated cardiac troponins; morpho-functional abnormalities on cardiac imaging; consistent tissue characterisation on cardiovascular magnetic resonance); in asymptomatic patients, two or more diagnostic criteria from different categories are required for diagnosis; coronary artery disease and other known causes should always be excluded. We included in the analysis only 409 patients with recorded levels of CRP (assessed by immunonephelometric method, Siemens Healthineers; normal levels < 6 mg/L) at the time of diagnosis. Clinical, electrocardiographic and laboratory characteristics and transthoracic echocardiographic (TTE) data were recorded at diagnosis and at each follow-up visit (planned at 6-month intervals, unless differently indicated by clinicians). Coronary artery disease was excluded by coronary angiogram or by cardiac computed tomography (patients aged < 35 years). Tissue characterization by cardiovascular magnetic resonance (CMR) was performed at diagnosis. Anti-heart auto-antibodies (AHA) were assessed by indirect immunofluorescence as previously described [14]. Endomyocardial biopsy (EMB) was performed according to international recommendations [1,15] and polymerase-chain-reaction (PCR) was performed on myocardial tissue to search for viral genome. The study was conducted according to the Helsinki Declaration and was approved by the local Ethics Committee (protocol number 0027841).

## 3. Statistical Analysis

Descriptive statistics were reported as median (IQR) for continuous variables and percentages (absolute numbers) for categorical variables. Wilcoxon and Chi-squared tests were performed to compare the distribution of continuous and categorical variables, respectively. Event-free (death or heart transplant, HTx) survival function was evaluated using the Kaplan–Meier estimator.

### RF Algorithm Development

To evaluate predictors of death/Htx, a random forest (RF) approach for survival data was employed. The RF [16] is a non-parametric ML algorithm not based on distributional or functional assumptions concerning the relationship of covariates to the response variable. The method is an ensemble learning tool developed for classification, regression and other predictive tasks that operate by constructing a forest of decision trees at training time. A single decision tree is a ML predictive tool that could be trained by performing repeated splitting procedures on the data. This process is repeated, on each derived subset, in a recursive manner (recursive partitioning). The recursion is completed when the subset splitting procedure no longer adds value to the prediction performance. The RF survival [17] is an extension of Breiman’s RF techniques applied to survival data, allowing efficient non-parametric analysis of time-to-event data, which has been proven as improving the learning performance as compared with base leaners [18].

As well as the improvement in the predictive capability of the model, an important reason for having chosen the RF algorithm to identify survival predictors in the current analysis is the low number of events at follow-up (*n* = 13) in our cohort. In such situations the estimation of more traditional multivariable Cox proportional hazard models is not recommended, in particular when the goal of the study is to explore potential factors associated with outcomes [19]. The RF algorithm has been shown to help in overcoming the limitations of more traditional statistical techniques, particularly when the ratio between the event number and covariates is below one [20].

The training of the algorithm was performed to identify the optimal mtry and nodesize tuning parameter according to the out-of-bag (OOB) error. The training was conducted considering 500 trees. The method underwent internal validation. To understand the importance of the covariates in predicting survival, a ranking of the covariates was provided according to the RF’s variable importance (VIMP) measure. The VIMP is a measure of the contribution of each variable to the model’s predictive accuracy. It represents the difference of the OOB prediction errors before and after each variable removal. The highest is the average increase of the OOB errors, the most important the variable is. A VIMP close to zero means that the variable does not contribute to the model’s predictive accuracy. Finally, to investigate the effect of the covariates found to be most important in predicting survival, the partial dependence plots showing the marginal effect of the variable on predicted survival were provided. The plot was performed considering the 3-year predicted survival probability since it roughly corresponds to the median of the follow-up time in our cohort. To evaluate the RF performance, the area under the curve (AUC), together with the 95% confidence interval (C.I.), was computed on the training and the OOB predictions. Analyses were performed with R software 4.1.0 with the packages rms, survival and randomForestSRC [19,21,22,23,24,25,26,27,28,29,30].

## 4. Results 

### 4.1. Patients’ Characteristics in the Overall Cohort

The patients’ features in the overall cohort are shown in Table 1. We included in the analysis 409 patients (74% male, mean age 37 ± 15 years), 129 (32%) with EMB-proven and 280 (68%) with clinically suspected myocarditis. PCR tested positive for viral genomes in 29 patients (22%); enterovirus (*n* = 4), herpes simplex virus 6 (*n* = 2), cytomegalovirus (*n* = 2) and Epstein-Barr virus (*n* = 2) were the most common viruses. Sixty-one patients (15%) had hypertension, 11 (3%) had diabetes; 21% had history of allergy and 9% were asthmatic. Nearly half of patients (*n* = 191) had a presumed viral infection in the 6 months preceding diagnosis. Fifty-six patients (14%) had a personal history of extra-cardiac auto-immune disease.

Half of the patients (223) complained of chest pain, and 10% and 5% reported palpitations and syncope, respectively. Almost all patients (97%) were in sinus rhythm at diagnosis; 7% had right bundle branch block, 3% had left bundle branch block and 3% had atrio-ventricular block at presentation. Biventricular dimensions on TTE at diagnosis were normal; left ventricular ejection fraction (LVEF) was mildly reduced (51 ± 13%), while right ventricular fractional area change was normal (42.1 ± 9.8%). Left ventricular (LV) volumes on LV angiogram were normal, while LV function was mildly reduced (53 ± 17%). CMR was performed in 328 patients (80%) at diagnosis; myocardial oedema was found in 190 (58%). Late gadolinium enhancement (LGE) was found in 288 (88%) patients, mainly with a non-ischemic pattern (mid-wall and/or subepicardial)(*n* = 257), while a diffuse LGE pattern was reported in 11 patients (4%) and an ischemic pattern in 21 patients (7%).

The median troponin I level at diagnosis was 4455 ng/L (IQR 438–13684). CRP levels were abnormal in 288 patients (70%). The median CRP level at diagnosis was 17 mg/L (IQR 4–54). Anti-heart auto-antibodies (AHA) tested positive in 174/334 (52%), anti-intercalated disk auto-antibodies (AIDA) in 116/335 (35%), anti-endothelial cell auto-antibodies (AECA) in 3/313 (1%), anti-nuclear auto-antibodies (ANA) and anti-mitochondrial auto-antibodies (AMA) in 37/321 (12%) and 3/264 (1%), respectively.

### 4.2. Clinical and Diagnostic Correlates and CRP Levels

Clinical and instrumental features at diagnosis in patients with and without abnormal CRP are shown in Table 1. Patients with abnormal CRP more frequently had a presumed viral infection in the 6 months preceding diagnosis (55% vs. 28%, *p* < 0.001), chest pain (60% vs. 42%, *p* < 0.001), clinically suspected myocarditis (74% vs. 26%, *p* < 0.001) and higher mean troponin I levels (6140 ng/L vs. 1150 ng/L, *p* < 0.001). Conversely, palpitations (7% vs. 17%, *p* = 0.002), syncope (3% vs. 10%, *p* = 0.002), arrhythmic presentation (22% vs. 4%, *p* < 0.001) and AHA positivity (*p* = 0.025) were more common in patients with normal CRP, who also had longer symptom duration (*p* < 0.001), and tended to have more advanced NYHA class at diagnosis (*p* = 0.054) (Central Illustration). There was no difference in CRP status (normal vs. abnormal) according to histological type in patients with EMB-proven myocarditis (*p* = 0.72).


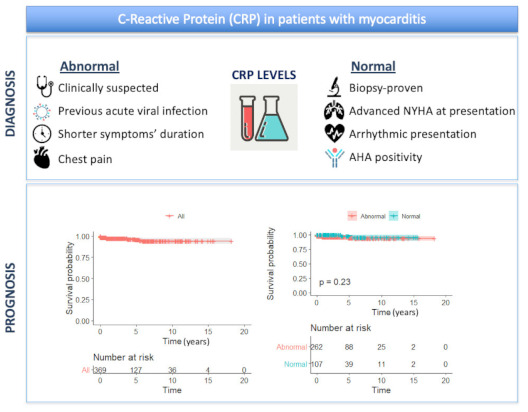


Central Illustration. Clinical and diagnostic role of C-reactive protein in myocarditis patients. Raised CRP identifies myocarditis patients with less severe clinical features, but shows negligible importance in predicting patients’ outcome.

The LV function on TTE, angiography and CMR did not differ according to CRP levels.

Myocardial oedema on CMR was more frequent in patients with abnormal CRP levels (*p* = 0.014), as well as LGE of the lateral wall (20% vs. 9%, *p* = 0.028). Conversely, septal LGE was more frequently noted among patients with normal CRP levels (9% vs. 3%).

### 4.3. Predictors of Survival

**Figure 1:**
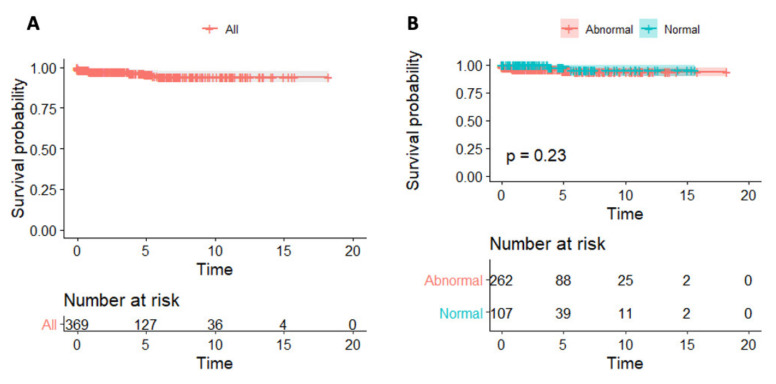
C-reactive protein and survival in patients with myocarditis. Kaplan–Meier survival curves in the entire studied cohort (**A**) and according to CRP levels (**B**).

Follow-up data were available for 369 patients (90%) for a median follow-up of 2.9 years (IQR 1.1–6.3). The pre-specified outcome of death/HTx was met in 13 patients (seven were transplanted and six died in end-stage heart failure), of whom 10 had biopsy-proven myocarditis. Overall survival was 97.6% at 1 year, 95.9% at 5 years and 94.1% at 10 and 15 years. There was no difference in survival with regards to CRP levels at any time interval (10-year survival 93.6% in patients with abnormal vs. 95.5% in patients with normal CRP, *p* = 0.23) (Figure 1) (Central Illustration).

The RF survival model was run on all covariates identified above. The performance of RF calculated on the training predictions was satisfactory (AUC 0.903, 95% C.I. 0.837–0.968) also when computed on the OOB predictions (AUC 0.790, 95% C.I. 0.699–0.881).

**Figure 2:**
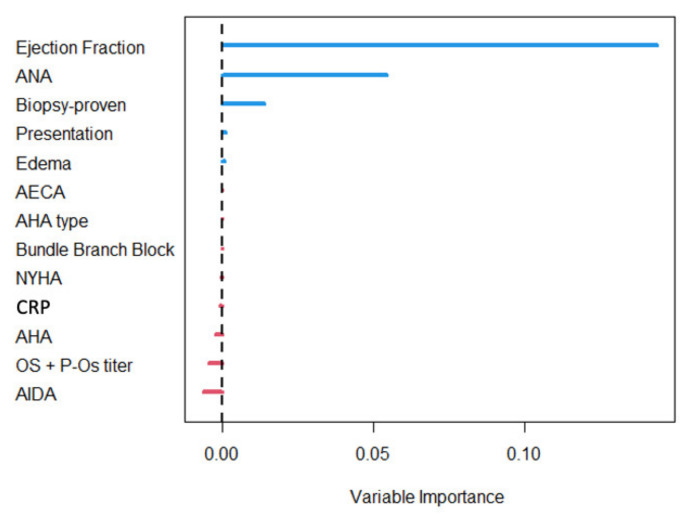
Variable importance plot according to the random forest algorithm. AECA, anti-endothelial cell auto-antibodies; AHA, anti-heart auto-antibodies; AIDA, anti-intercalated disk auto-antibodies; ANA, anti-nuclear auto-antibodies; CRP, C reactive protein; OS, organ specific.

**Figure 3:**
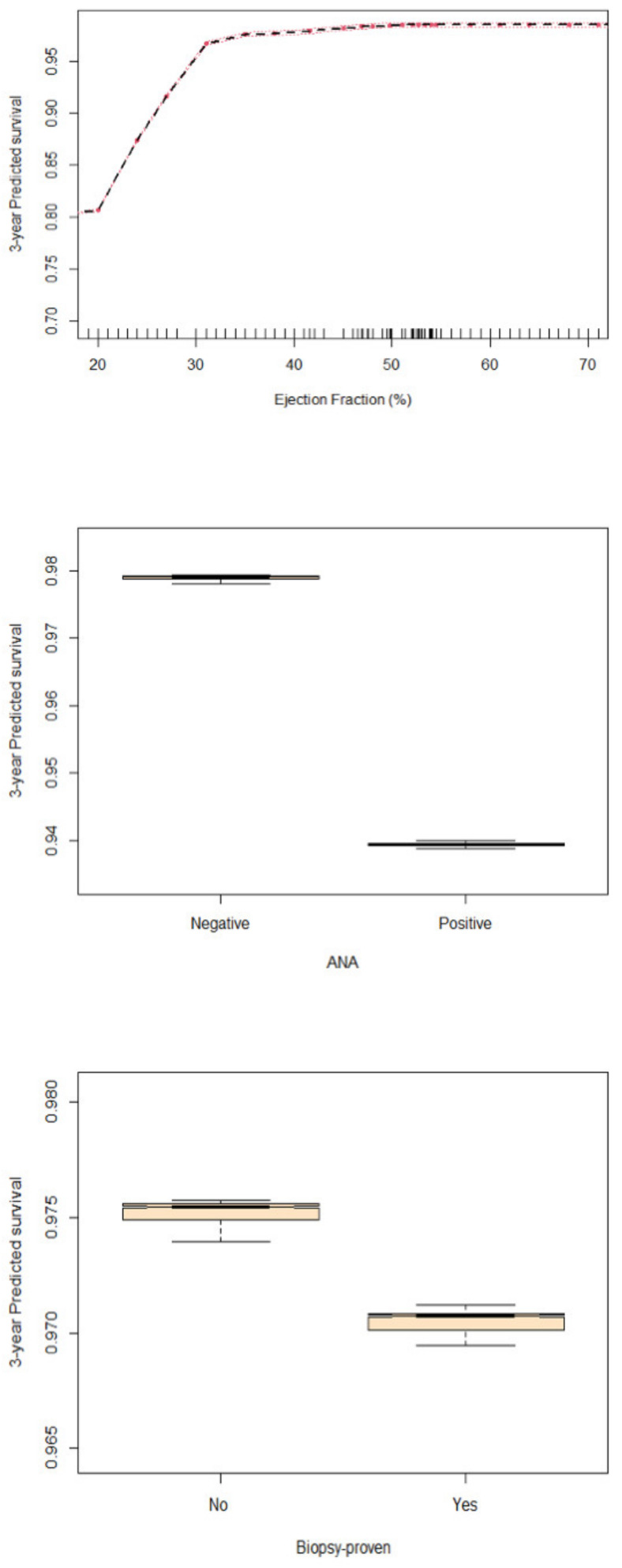
Plot of the marginal effect of left ventricular ejection fraction (LVEF), anti-nuclear auto-antibodies (ANA) and biopsy proven status on 3-year predicted survival probability.

According to the VIMP4 measure (Figure 2), the strongest survival predictor over the entire follow-up was LVEF, followed by ANA positivity and biopsy-proven status, the last two with a lower impact on the predictive accuracy of the model (Figure 2). The partial dependence plot (Figure 3) of LVEF shows that the predicted survival probability was higher for higher ejection fraction values, with a plateau for LVEF values over 30%. The predicted survival probability was lower for patients with positive ANA (Figure 3) and with biopsy-proven myocarditis (Figure 3). The limited contribution of CRP in characterizing patient survival was confirmed in the RF analysis (VIMP value −0.0001).

## 5. Discussion

### 5.1. Diagnostic Role of CRP in Myocarditis

Data regarding the diagnostic role and correlates of abnormal CRP levels in myocarditis are lacking. To the best of our knowledge, for the first time we report that the frequency of abnormal CRP levels at diagnosis is high, up to 70% from a large consecutive cohort of patients with biopsy-proven and clinically suspected myocarditis, strictly defined according to the 2013 ESC criteria [1].

In our cohort, CRP failed to identify patients with worse clinical features at diagnosis, since these patients had normal CRP levels. Our findings are in keeping with the 2013 ESC consensus on myocardial and pericardial diseases, clarifying that increased inflammatory markers have only an ancillary diagnostic role in myocarditis [1].

We found higher CRP levels in male patients with clinically suspected myocarditis, a history of acute viral infection in the 6 months preceding diagnosis, shorter symptoms’ duration, and higher chest pain frequency and troponin I levels. Thus, higher CRP levels identified patients with clinically suspected myocarditis with infarct-like presentation, which are known to have better functional status and a more favourable disease course [2,3,31]. Conversely, we found lower CRP levels among patients with biopsy-proven myocarditis, arrhythmic presentation, more advanced NYHA class at presentation and a higher likelihood of AHA positivity. This suggests that in myocarditis autoimmune features are associated with a worse disease course [2,31,32,33], as recently shown by our group [34]. A role of innate immunity, i.e., the inflammasome, in the development of myocarditis has been suggested [35], leading to proposing a role for anti-interleukin-1 treatment. Anti-interleukin 1 immunomodulatory agents targeting the inflammasome have been shown to reduce cardiovascular events in coronary atherosclerosis patients with abnormal high sensitivity CRP [36], to improve left ventricular remodelling in those with ST elevation myocardial infarction [37] and to treat patients with idiopathic recurrent acute pericarditis (IRAP) [38,39]. The lower CRP values that we found in myocarditis patients with worse outcomes are in keeping with an established major role for adaptive response, i.e., autoimmunity, rather than innate immunity [1,2,14,40,41].

While the role of systemic inflammation is established in ischemic heart disease, holding a central place in the initiation and progression of atherosclerosis [5,6,7], less is known about its role in non-ischemic cardiomyopathies. A previous study on 59 patients with left ventricular dysfunction of different aetiology showed significantly higher CRP levels in patients with ischemic left ventricular dysfunction than in those with non-ischemic left ventricular dysfunction, and that acute myocardial infarction is associated with higher CRP levels than chronic left ventricular dysfunction [42].

CRP levels have also shown to reflect tissue damage in many diseases [6]; a large study on 610 patients with systemic lupus erythematosus found an association between increased CRP levels and different types of organ damage, including myocarditis. However, no endomyocardial biopsy data were shown; therefore, in the absence of histological confirmation such association remains unproven [43].

New computed tomography-based software, enabling the assessment of coronary inflammation by means of a higher pericoronary fat attenuation index (pFAI), has been used for patients with coronary artery disease of different severity [44]. Using this novel tool, we explored the potential presence of coronary inflammation in clinically suspected myocarditis with infarct-like presentation; our findings suggest that pFAI may be a non-invasive marker of non-atherosclerotic, infectious or immune-mediated “endothelialitis” [45]. However, so far only weak correlations between organ-specific heart inflammation, i.e., myocarditis and endothelialitis, and systemic inflammation (as assessed by CRP and circulating serum cytokines) have been shown, both in ischemic and non-ischemic cardiomyopathies [44,46,47].

In spite of the still debated diagnostic role in myocarditis, CRP is elevated in the majority of patients with acute pericarditis where it is used to monitor disease activity and to establish the appropriate length of anti-inflammatory treatment [48]. Normalization of CRP levels within one week has also been shown to identify patients with lower risk of recurrent pericarditis [48]. In our study no patient had concomitant pericarditis.

### 5.2. CRP and Myocarditis Prognosis

In our cohort death/Htx was observed in only 13 patients (3.5%; seven were transplanted and six died for end-stage heart failure), of whom 10 had biopsy-proven myocarditis. The low recurrence of adverse events in our cohort can be explained by the overall only mildly impaired LVEF and the high prevalence of clinically suspected myocarditis (68% of patients), known to have a more benign disease course [1,3,34].

Overall survival did not differ based on CRP levels, which did not contribute to the predictive accuracy of the model for outcome prediction. Our findings are in keeping with a recent study on a large cohort of idiopathic heart failure patients with immunohistochemically defined myocardial inflammation on EMB; myocardial inflammation was associated with higher CRP and troponin I levels, but CRP failed to predict prognosis [8]. Conversely, Kaneko et al. analysed the prognostic role of CRP in 31 patients with biopsy-proven lymphocytic myocarditis and found significantly higher CRP levels among the five dead patients [49]; however these five patients also had significantly lower LVEF on echocardiogram (29% vs. 49%). Clearly the low numbers of patients in this study did not allow the authors to perform a multivariable analysis. Thus, an independent prognostic role of CRP remains unproven, particularly in consideration of the established and predominant independent negative prognostic role of reduced LVEF in myocarditis, which per se might account for the mortality association [34,41,50,51,52,53,54,55,56]. In another study on 188 patients with dilated cardiomyopathy of unspecified aetiology, CRP levels were higher among the 49 patients who died as compared to those alive after a 5-year follow-up; once again, in the absence of rigorous diagnostic work-up for clinically suspected or biopsy proven myocarditis and of multivariable analysis, the role of CRP in this study remains undefined [57].

In the present study, the strongest survival predictor was LVEF, followed by ANA positivity and biopsy-proven status. The strong prognostic role of LVEF has been documented in all cardiovascular diseases, irrespective of aetiology, and is in keeping with previous studies and trials on myocarditis, which showed significantly lower survival in patients with reduced LVEF [41,50,51,52,53,54,55,56], especially before the introduction of immunosuppression [3,34]. In keeping with previous data from the literature [8] we found no difference in biventricular function on TTE according to CRP levels. We also found no difference in biventricular function on CMR and no difference in LGE prevalence according to CRP levels, which is in keeping with previous studies on clinically suspected myocarditis [58]. The lack of association of CRP with biventricular function indexes found here and in previous work reinforces the lack of prognostic value of CRP, since biventricular function is an established independent predictor in clinically suspected and in biopsy-proven myocarditis [1,3,34,41,50,51,52,53,54,55,56]. The prognostic role of biopsy-proven status is likely to reflect selection bias in performing EMB in patients with worse clinical and diagnostic features, as well as the predominance of autoimmune virus-negative myocarditis in biopsy-proven patients [41]. In keeping with the worse outcome in autoimmune myocarditis, in this study and in another recent publication from our group [34] we found a prognostic role of anti-nuclear autoantibodies. In another recent study on systemic cutaneous sclerosis, we found that serum AHA were associated with cardiac involvement and increased risk of cardiac death [33].

For the first time, a RF approach for survival data was employed to evaluate predictors of death/Htx. Our RF approach is a tree-based machine learning algorithm, increasingly used in the clinical setting [9,10,11,12,13]. The main advantage of this algorithm is that it has the capability to identify survival predictors in spite of a low number of events at follow-up (*n* = 13 in our cohort), which prevents risk estimation by more traditional multivariable Cox proportional hazard models. In other terms, the RF algorithm overcomes the limitations of more traditional statistical techniques, particularly when the prevalence of the event of interest is low, since it allows the detection of complex relationships between the outcomes and the covariates, even though a high number of predictors are evaluated in front of a low number of events [16,17,18,19,20,21,22,23,24,25,26,27,28,29,30].

## 6. Limitations

The main limitations of our study are the retrospective design and the low number of events at follow-up. The use of the RF approach may have contributed to partially overcoming such limitations. Nevertheless, the lack of an external cohort to validate the machine learning model still recommends cautiousness in generalizing our findings. In addition, the high frequency of clinically suspected myocarditis may have blunted the prognostic role of CRP. The identification of biopsy features predicting outcome was beyond the scope of this study, since rare myocarditis forms, such as giant cell myocarditis [59,60], were underrepresented in this cohort. However, in previous publications on our larger patient cohort, we found that giant cell myocarditis is associated with worse prognosis compared to the other histological types [34,41].

## 7. Conclusions

C-reactive protein was elevated in the majority of myocarditis patients but it should still be used as an ancillary feature, rather than a specific diagnostic and prognostic biomarker, as it did not identify myocarditis patients with worse clinical features. In our cohort of clinically suspected and biopsy-proven myocarditis, CRP levels at diagnosis did not contribute to the predictive accuracy of the outcome using a machine-learning RF prediction model. The main death/Htx predictors were reduced LVEF, biopsy proven diagnosis and positive antinuclear autoantibodies.

## Figures and Tables

**Table 1:** Patients’ characteristics.

	All *n* = 409	Abnormal CRP Levels *n* = 288	Normal CRP Levels *n* = 121	Missing Data	*p*-Value
Age, years	37 ± 15	36 ± 15	39 ± 16	-	0.23
Sex, male	301 (74)	224 (78)	77 (64)	-	0.003
EMB-proven myocarditis	129 (32)	74 (26)	55 (45)	-	<0.001
Clinically suspected myocarditis	280 (68)	214 (76)	66 (24)	-	<0.001
Hypertension	61 (15)	39 (14)	22 (18)	-	0.23
Diabetes	11 (3)	7 (2)	4 (3)	-	0.62
Immune-mediated diseases	56 (14)	37 (13)	19 (16)	2	0.46
Acute viral infection in the preceding 6 months	191 (47)	158 (55)	33 (28)	3	<0.001
Symptoms duration, months	0.5 (0.06–3)	0.2 (0.03–1)	3 (0.3–18)	257	<0.001
Chest pain	223 (55)	172 (60)	51 (42)	-	<0.001
Palpitations	41 (10)	20 (7)	21 (17)	-	0.002
Syncope	20 (5)	8 (3)	12 (10)	-	0.002
Symptoms variation in the 12 months preceding diagnosis	66 (16)	39 (14)	27 (22)	-	0.029
Clinical presentation, infarct-like	25 (6)	19 (7)	6 (5)	-	0.53
Clinical presentation, heart failure	68 (17)	46 (16)	22 (18)	-	0.58
Clinical presentation, arrhythmias	39 (10)	12 (4)	27 (22)	-	<0.001
NYHA class II-IV	61 (15)	37 (12)	24 (21)	1	0.054
LV heart failure at presentation	69 (17)	47 (16)	22 (18)	-	0.65
RV heart failure at presentation	26 (6)	18 (6)	8 (7)	-	0.89
Troponin I, ng/L	4455 (438–13684)	6140 (1148–15298)	1150 (12–6030)	-	<0.001
AHA positivity	174 (52)	112 (48)	62 (61)	75	0.025
AIDA positivity	116 (35)	75 (32)	41 (41)	74	0.25
AECA positivity	3 (1)	2 (1)	1 (1)	96	0.91
ANA positivity	37 (12)	26 (11)	11 (11)	88	0.95
LVEF Angio, %	53 ± 17	54 ± 16	53 ± 18	-	0.87
LVEDVi Echo, mL/m^2^	68 ± 24	67 ± 22	70 ± 28	60	0.69
LVEF Echo, %	51 ± 13	51 ± 13	50 ± 14	27	0.65
RVEDA Echo, cm^2^	20.8 ± 4.7	20.7 ± 4.3	21.0 ± 5.7	205	0.96
RVFAC Echo, %	42.1 ± 9.8	42.2 ± 10.4	41.9 ± 8.5	219	0.73
Mitral regurgitation Echo, moderate-severe	34 (10)	26 (10)	8 (9)	78	0.84
LVEDVi CMR, mL/m^2^	88 (78–104)	88 (79–102)	90 (74–106)	-	0.83
RVEDVi CMR, mL/m^2^	82 (74–94)	84 (74–95)	80 (70–91)	-	0.22
LVEF CMR, %	57 (52–62)	57 (53–62)	59 (51–62)	-	0.69
RVEF CMR, %	58 (54–63)	58 (53–62)	59 (55–65)	-	0.12
Presence of myocardial edema on CMR	190 (58)	145 (61)	50 (47)	81	0.014
Presence of myocardial LGE on CMR	288 (88)	210 (90)	78 (84)	83	0.17
Lateral wall LGE	37 (17)	31 (20)	6 (9)	190	0.028

Data are expressed as mean ± SD, median (IQR) and *n* (%). AID, auto-immune diseases; AECA, anti-endothelial cell auto-antibodies; AHA, anti-heart auto-antibodies; AIDA, anti intercalated disk auto-antibodies; ANA, anti-nucleus antibodies; CMR, cardiovascular magnetic resonance; EDA, end-diastolic area; EMB, endomyocardial biopsy; LGE, late gadolinium enhancement; LVEDVi, indexed left ventricular end-diastolic volume; LV, left ventricular; LVEF, left ventricular ejection fraction; NYHA, New York Heart Association; RV, right ventricular; RVEDA, right ventricular end-diastolic area; RVEDVi, indexed right ventricular end-diastolic volume; RVEF, right ventricular ejection fraction; RVFAC, right ventricular fractional area change.

## Data Availability

Data will be available upon reasonable request to the Corresponding Author.
